# A feasibility cluster randomised controlled trial of a preschool obesity prevention intervention: ToyBox-Scotland

**DOI:** 10.1186/s40814-019-0521-7

**Published:** 2019-11-09

**Authors:** Stephen Malden, John. J. Reilly, Ann-Marie Gibson, Farid Bardid, Carolyn Summerbell, Marieke De Craemer, Greet Cardon, Odysseas Androutsos, Yannis Manios, Adrienne Hughes

**Affiliations:** 1 Physical Activity for Health Group, School of Psychological Sciences and Health, University of Strathclyde, Graham Hills Building, 40 George Street, Glasgow, G1 1XP UK; 2 School of Education, University of Strathclyde, Glasgow, UK; 3 Department of Movement and Sports Sciences, Ghent University, Ghent, Belgium; 4 Department of Sport and Exercise Sciences, Durham University, Durham City, UK; 5 Department of Nutrition and Dietetics, School of Health Science and Education, Harokopio University, Athens, Greece

**Keywords:** Childhood obesity, Feasibility, Physical activity, Sedentary behaviour, Prevention

## Abstract

**Background:**

High levels of childhood obesity have been observed globally over the last three decades. Preschools are promising settings to implement obesity prevention interventions in the early years. The aim of this study was to test the feasibility of a cluster randomised controlled trial of the ToyBox-Scotland preschool obesity prevention intervention.

**Methods:**

Six preschools in predominantly deprived areas of Glasgow, UK, were randomised to either the ToyBox intervention (*n* = 3) or usual curriculum control group (*n* = 3). The intervention ran for 18 weeks from March–June 2018, and consisted of practitioner-led physical activity and sedentary behaviour sessions in preschools, with an additional interactive home component. Primary outcome measures were intervention fidelity, recruitment rates, attrition rates, and compliance with trial procedures. Secondary outcomes were body mass index (BMI) *z*-score, bioelectrical impedance analysis (BIA), objectively measured physical activity and sedentary time via activPAL accelerometer, and parent-reported home eating, snacking, and water consumption.

**Results:**

The preschool component of the intervention was implemented with high fidelity (64%), while the home component was implemented with low fidelity (41%). A cluster-level recruitment rate of 10% was achieved, and the individual-level recruitment rate was 18% (42/233 children, mean age 4.4 years; 17 girls). The attrition rate was 14%, and compliance rates varied considerably by the outcome. Compliance was highest for BMI (86%), while 19% of the sample returned valid accelerometer data for both baseline and follow-up and the parental questionnaire response rate was 23%. Both intervention and control groups showed small increases in BMI *z*-scores at follow-up of 0.02 and 0.06, respectively. Both groups had small decreases in physical activity and increases in sedentary time at follow-up.

**Conclusions:**

Before progression to an effectiveness trial, additional procedures should be considered to improve recruitment rates, compliance with outcome measures, and implementation of the home-based component of the ToyBox-Scotland intervention.

**Trial registration:**

ISRCTN12831555

## Background

High levels of childhood obesity are evident globally [1], with obesity in the early years being linked to elevated total and low-density lipoprotein (LDL) cholesterol in children as young as 3 years [2]. While the causes of childhood obesity are multifactorial, research has demonstrated causal links between excess weight and energy balance-related behaviours such as physical activity (PA), sedentary behaviour (SB), and diets high in fat and refined sugars [3, 4].

Preschools offer a potentially effective setting to address obesity prevention, and a number of interventions have targeted such settings with varying levels of success [5–7]. Specifically, multicomponent interventions which target PA, SB, and diet both in the preschool and home environment tend to show the most promise with regards to improving energy-balance-related behaviours and preventing obesity in young children [8, 9].

One such intervention is called ToyBox, which employs teacher-led sessions to target energy-balance related behaviours at preschool, while behaviours in the home environment are targeted using informative materials for parents [10]. The intervention, when tested in six countries across Europe, led to significant improvements in water consumption [11], PA, SB [6], and family-related determinants of unhealthy snacking [12]. The intervention has subsequently been adapted for use in other European countries, Malaysia [13], and most recently in Scotland [14], where context-specific adaptations were made to the intervention content and delivery to suit the social and cultural needs of Scottish preschools. However, prior to testing an intervention in an effectiveness trial, the UK Medical Research Council recommends that a feasibility study should be conducted first as it is considered an integral aspect of intervention development and evaluation [15]. Therefore, the aim of this study was to test the feasibility of a cluster randomised controlled trial (RCT) of the ToyBox-Scotland preschool obesity prevention programme to inform the design of a future full-scale RCT.

## Methods

### Study design

This study was designed in accordance with the CONSORT statement’s extension to randomised pilot and feasibility trials [16]. This study had a cluster RCT design consisting of an intervention group (three preschools) and a control group (three preschools). As this was a feasibility study, no sample size calculation was undertaken. As all participating preschools were similar in size and demographics, no matching was undertaken prior to randomisation. An independent researcher was presented with six identical envelopes by a member of the research team not involved in data collection or analysis. Each envelope contained the name of the participating preschools. They were then instructed to select three envelopes at random to be control preschools. The remaining envelopes were assigned to the intervention group. Data were collected between January and June 2018. This study was approved by the University of Strathclyde’s School of Psychological Sciences and Health Ethics Committee.

### Setting, sampling, and participants

Glasgow is the largest urban area in Scotland and is one of the most socioeconomically deprived areas in Western Europe, with over a third of the cities’ children estimated to be living in poverty [17]. A Glasgow City Council representative contacted a convenience sample of all local authority preschools in the Glasgow City area via email to seek expressions of interest to participate (*n* = 112). Eleven preschools expressed an interest to participate in the study, of which 6 were selected based on similarities in demographics, size, and socio-economic status (SES). Head teachers at participating preschools were visited by the study manager and provided with information sheets and consent forms, which they distributed to parents/caregivers of all 3–5-year-old children at their preschools. Children were excluded from the study if they had a health condition that would significantly limit their ability to participate in the intervention or if parental consent was not provided. The intervention was delivered to all 3–5-year-old children in the intervention preschools. All six preschools received £200 after the completion of the study to offset any participation costs.

### Intervention

Prior to the commencement of the present study, the original ToyBox intervention [10] was adapted to suit the Scottish preschool setting. The process of adaptation is described in detail elsewhere [14]. Briefly, alterations to the number of PA and SB sessions were made to reflect the focus on child-led learning in Scottish preschool practice and classroom manuals were re-written to reflect the language used in the Scottish education system. Additional adaptations included the removal of the preschool-based eating/snacking and water consumption components and the addition of more interactive parent/child activities to address energy-balance behaviours (i.e. eating/snacking, water consumption, PA, and SB) in the home environment. All adaptations were undertaken using a co-creation approach [18], whereby relevant stakeholders and an experienced early years’ practitioner assisted the research team with the adaptation process.

Preschools receiving the ToyBox-Scotland intervention were provided with a ~ 2.5 h practitioner training session prior to the intervention. Preschools received a box with additional classroom materials such as kangaroo hand puppets and classroom activity guides. Classroom activity guides offered detailed instructions on the delivery of PA and SB sessions, and the setup of the classroom environment to encourage PA and active play and to reduce SB. Practitioners were given autonomy to deliver the intervention throughout the day when time allowed, but were encouraged to deliver activities for a total of 1 h per week, and gradually increase this as the intervention progressed. Parents received a sticker wallchart and bi-weekly activity packs containing tip cards, newsletters, interactive games and sticker incentives to award to their child after they completed each of the home-based activities. The intervention was delivered for 18 weeks, where PA and SB were targeted in both the preschool and home environment, and eating and water consumption was targeted in the home environment, as detailed in Table 1.

**Table 1:** Intervention structure for ToyBox-Scotland

	First focus							Repetition								
	3 weeks		3 weeks		3 weeks		3 weeks		2 weeks		2 weeks		1 week		1 week	
Preschool	PA		SB		PA		SB		PA		SB		PA		SB	
Home	WC	PA	ES	SB	WC	PA	ES	SB	WC	PA	ES	SB	WC	PA	ES	SB

*ES* eating and snacking, *PA* physical activity, *SB* sedentary behaviour, *WC* water consumption

### Procedures and outcomes

Participants were measured at two time-points by one researcher (SM) and a fieldwork assistant, who were both trained in the measures. Baseline assessment was undertaken in late January/early February 2018, with follow-up measurement taking place 15–17 weeks later. An early years’ practitioner at each preschool was present for all data collection procedures to prepare and accompany children through data collection and assist with any issues. Although parental consent was collected for all participating children, child assent was obtained from each child on the measurement day, and children who did not want to participate in any of the data collection procedures were not obliged to do so. The primary outcome measure for this study was the feasibility of the intervention and trial. Therefore, the primary outcomes of interest were recruitment rates, attrition rates, implementation fidelity and compliance rates with data collection procedures. A number of secondary outcomes were also assessed, detailed below.

### Implementation fidelity

Implementation fidelity refers to the extent to which an intervention is implemented as intended by those who developed it [19]. Fidelity was assessed in both the preschool and home environments using the following methods:

#### Preschool component

In order to assess implementation fidelity at preschools, practitioners were supplied with a monthly logbook for the duration of the programme, which was adapted from the original ToyBox study logbook [20]. For each month that the intervention was delivered (*n* = 4), practitioners completed five-point Likert scales, which assessed the extent to which the components of the intervention were delivered. Namely, changes to the classroom environment, children performing health behaviours, and classroom experiences.

#### Home component

Practitioners recorded how many eligible children were supplied with home activity packs each month, while parents/caregivers received a post-intervention questionnaire (Additional file 1). This questionnaire comprised yes/no questions and 5-point Likert scales, with questions designed to identify to what extent the parents/caregivers received and engaged with the intervention materials at home.

### Secondary outcome measures

#### Body mass index

Height and weight were measured by the same researcher with children wearing light clothing and with shoes removed. Height was measured using a stadiometer (Marsden, UK) to the nearest 0.1 cm, and weight was measured using an electronic scale (Tanita, Amsterdam, Netherlands) to the nearest 0.1 kg. Measurements were conducted in a private meeting room, with children measured in small groups of 3–4 at a time. Only the researcher was able to see the readings. Both height and weight measurements were taken twice and the average calculated. Body mass index (BMI) *z*-scores were calculated from the height and weight data using standardised methods [21]. Children aged ≥ 4 years were categorised using UK90 growth reference charts [22], while the WHO growth reference was used to calculate *z*-scores for 3-year-olds [23]. Children < 85th percentile were classified as normal weight, ≥ 85th percentile as overweight, and ≥ 95th percentile as obese.

#### Objectively measured PA

PA was measured objectively using the activPAL accelerometer (model ActivPAL3; PAL Technologies Ltd., Glasgow, UK). The activPAL is a small wearable device that is attached to the front of the mid-thigh and measures postural information, which can be categorised into sitting/lying, standing and moving/stepping activity [24]. Once attached, the device can be worn continuously for 7–10 days. Participants were fitted with the activPAL by assisting early-years practitioners under the instruction of the researchers. Parents were instructed to leave the activPAL on for seven consecutive days, with 3 days wear time [25] considered valid for this study.

#### Body composition

Supine arm-to-leg bioelectrical impedance analysis (BIA) was used to measure fat mass and fat-free mass with the Bodystat 1500 (Bodystat Ltd., Douglas, Isle of Man). Measures were taken twice and the average was calculated. A full description of the procedures and formulae to use with this age group is available elsewhere [26].

#### Objectively measured SB

The activPAL was used to assess sedentary time during waking hours using the same procedure as PA described above [27]. Periods of nighttime sleep were differentiated from waking sedentary time by studying the raw data files to determine when no significant changes in axis of movement (from sitting/lying to standing) were detected from one 24-h period to the next, as such observations indicate the participant is asleep during these times.

#### Home eating, snacking, water consumption, and screen time

The Primary Caregiver Questionnaire (PCQ) and the Food Frequency Questionnaire (FFQ) used in the original ToyBox study were adapted for use in the present study. Specifically, the number of questions in each were reduced as recommended by stakeholders during development meetings (questions related to maternal/post-natal nutrition were removed from the FFQ and family history questions removed from the PCQ). The questionnaires required parents to provide information on their children’s fruit/vegetables, confectionary, water, and sugar-sweetened beverage consumption in addition to the use of screen devices and sleep patterns. Questionnaires were supplied to preschools by the research team in paper format and were then distributed to participating parents when they collected their children by preschool staff. Full details regarding the development, validity, and test-retest reliability of the questionnaires are reported elsewhere [28–30].

### Analysis

In order to assess fidelity of implementation in this study, scoring systems used by Verloigne et al. [31] and Pinket et al. [32] were adapted and used to assign codes to participant’s logbook and questionnaire responses that indicated the level of implementation. For dichotomous items, a positive response (yes) was coded as 1, while a negative response (no) received a 0. For Likert scale items, a response of either 4 (agree/often) or 5 (strongly agree/always) was coded as 1, while all other responses (1–3, strongly disagree/never; disagree/not often; neither agree nor disagree/sometimes) were coded as 0. Total implementation fidelity scores of 72 and 11 were available for practitioners and parents, respectively. Accelerometer data were entered into PAL analysis software and mean daily time spent in PA, step count, sedentary time, and sleep were computed for all devices which met the 3-day valid wear-time cut-off. To calculate and categorise participant’s weight status from the height and weight measurements, data was entered into the LMS Growth add-in for Microsoft Excel to generate *z*-scores and percentile scores. As this is a feasibility study, the use of inferential statistics and effectiveness testing is not recommended due to the small sample size and the preliminary nature of the outcomes measured [33]. Instead, descriptive statistics were used to assess feasibility parameters such as fidelity of implementation, recruitment, retention and attrition rates from baseline to follow-up, presented as proportions. High, medium, and low fidelity was classified as an overall implementation score of ≥ 60%, ≥ 50 < 60%, and < 50% respectively, following recommendations from similar studies [19]. For the secondary outcomes, means ± standard deviations are presented, with the mean change from baseline to follow-up for each outcome calculated along with 95% confidence intervals where appropriate. Process evaluation data (e.g. teacher logbooks and parental feedback surveys) were analysed prior to outcome data, as recommended by current guidelines on process evaluation [34].

## Results

### Feasibility of trial recruitment and retention

**Fig. 1:**
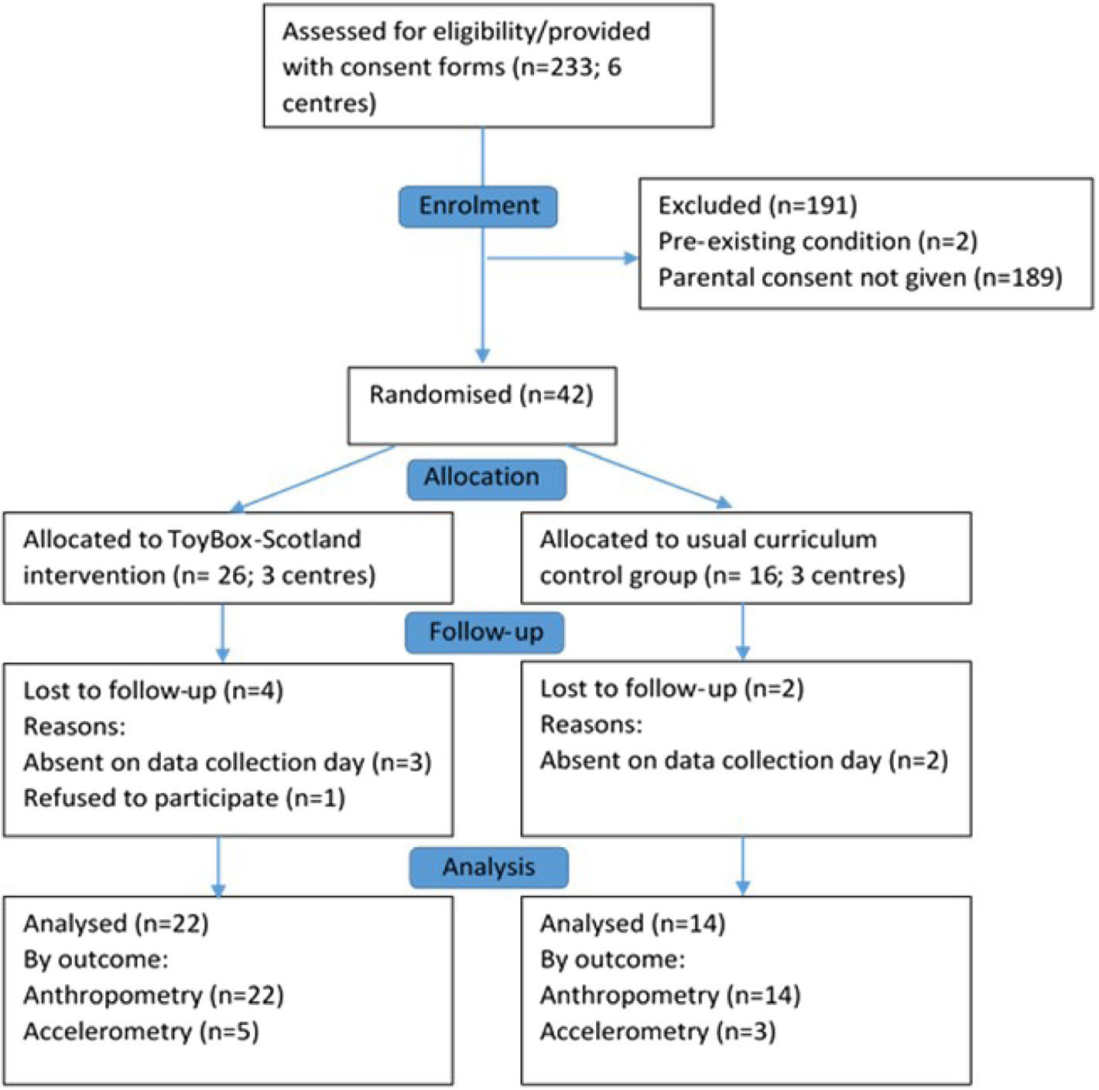
CONSORT flow diagram detailing trial recruitment and retention for ToyBox-Scotland

Eleven out of 112 preschools responded positively to an invitation to take part in the study (cluster-level response rate = 10%). A total of 233 consent forms were distributed, of which 42 children (mean age 4.4 ± 0.46 years; 17 girls) provided parental consent and completed baseline assessment (individual-level recruitment rate = 18%) before preschools were randomised to the ToyBox-Scotland intervention arm (3 centres; *n* = 26; 10 girls) or the usual curriculum control arm (3 centres; *n* = 16; 7 girls). See Fig. 1 for CONSORT flow diagram.

### Intervention fidelity

All intervention preschools returned complete logbooks for the 4 month study period. Overall, the intervention was implemented with high fidelity across the three intervention preschools (64%), with one preschool implementing with medium fidelity (52%), and two with high fidelity (79% and 61%). Intervention components relating to PA were generally implemented with higher fidelity than SB components (Table 2). Twenty-six parents returned post-intervention feedback surveys, of which seven were incomplete and excluded (19/125; 15% response rate). The overall implementation score for the home component of the intervention was low (41%) based on post-intervention survey responses. Specific preschool implementation scores from practitioners’ logbook data are detailed in Table 2.

**Table 2:** Implementation fidelity score logbook items and responses

Component	Logbook question	Scoring and results (% coded as 1 over the 4 months)			
		PS A (%)	PS B (%)	PS C (%)	Overall (%) (fidelity score)
Preschool environment	Were the number of chairs in the classroom reduced to encourage standing play?*	0	0	0	0 (low)
	Was equipment and space appropriately arranged for physical activity sessions every day of the week?*	100	50	100	83 (high)
	Was the classroom appropriately arranged for movement breaks every day of the week?*	100	75	0	58 (med)
	Were any movement corners set up and made available to the children?*	75	0	0	25 (low)
Children performing the health behaviours	Did you regularly remind children to drink water?*	100	100	100	100 (high)
	Did you remind children to drink water after they have been active?*	100	100	100	100 (high)
	Did you remind children to bring healthy snacks from home (or remind the catering service/canteen to provide healthy snacks to children?)*	100	100	100	100 (high)
	How much time did you devote to physical activity sessions on an average weekly basis this month? ^+^	100	75	100	92 (high)
Classroom experiences	Did you implement the classroom experiences for physical activity as described in the manual?*	100	50	100	83 (high)
	Did you devote on average at least 1 h per week to the classroom activities for physical activity as described in the manual?*	100	50	100	92 (high)
	Did you devote on average at least 1 h per week to the classroom activities for sedentary behaviour as described in the manual?*	50	0	0	17 (low)
	Which classroom activity(ies) regarding physical activity did you implement this month? ^+^	100	50	50	67 (high)
	How many of the little kangaroo stories for physical activity did you use this month? ^+^	19	0	25	11 (low)
	How many of the little kangaroo stories for sedentary behaviour did you use this month? ^+^	8	0	0	3 (low)
	Which classroom activity(ies) regarding sedentary behaviour did you implement this month? ^+^	100	25	0	42 (low)
Delivery of home materials and engagement with parents	Did you provide parents with the pre-prepared home activity packs when these were delivered to the nursery?*	100	75	100	92 (high)
	Estimate the number of parents to whom you directly delivered programme materials. If you did ^+^ (total 125 children)	100	85	100%	95 (high)
	Estimate the number of parents for whom you spent time to explain the purpose of the material and encourage them to follow the recommendations of the material ^+^ (total 125 children)	11	7	15	12 (low)
	Total aggregate scores (% responses coded as 1. Total available points = 72	79	52	61	Overall score = 64

This form was repeated four times, once for each month the intervention was delivered

*Scoring determined by 5-point scale, “*1* = never, *2* = not often, *3* = sometimes, *4* = often, *5* = always” ≥ 4 = 1; ≤ 3 = 0

^+^Scoring determined by a “yes/no” response or a numerical response. *Yes* = 1; *no* = 0. Numerical responses equate to *≥ 60%* = 1; *< 60%* = 0. *PS* = preschool

### Participation in outcome measures

#### Anthropometry

Eighty-six percent (36/42) of participants provided valid height and weight measurements at baseline and follow-up. Five children were absent on the follow-up data collection day, and one did not want to participate.

#### Body composition

Six children out of 42 (14%) adequately complied with the BIA protocol at baseline. However, the readings from these were not valid as children did not adhere to the protocol and the use of BIA was not carried forward to follow-up.

#### Accelerometry

Fifty-two percent of the participants provided valid accelerometer data at baseline (*n* = 22). Reasons for invalid measurement were as follows: device malfunction (*n* = 8); removed before valid wear-time due to skin irritation (*n* = 7); device loss (*n* = 5). Only participants who supplied valid data at baseline were fitted with an accelerometer at follow-up. Nineteen percent of the original sample returned valid accelerometer data at follow-up. Reasons for loss of data at follow-up were removed before valid wear time cut-off (no specific reason provided; *n* = 5); device lost (*n* = 4); child absent on data collection day (*n* = 5).

#### Parental questionnaires (demographics/family history, dietary habits, screen time)

Early years practitioners distributed the PCQ and FFQ to the parents/caregivers of all participating children at baseline (*n* = 42). Twenty-three percent of parents returned completed questionnaires for both baseline and follow-up (*n* = 10).

### Behavioural and health outcomes

For the 22 participants that provided valid accelerometer data at baseline, mean daily minutes spent in PA was 163 (30) and 151 (40) for the intervention and control groups respectively (mean daily steps of 11,437 (2351) for the intervention group, and 10,827 (2895) for the control group). The intervention group spent an average of 420 (72) min/day sedentary, and the control group spent 396 (72) min sedentary. Table 3 summarises the results for participants that completed measurement at baseline and follow-up. Small increases in BMI *z*-score were observed for both groups; however, the increase was larger in the control group. Both groups showed reductions in mean daily minutes spent in PA and daily steps from baseline to follow-up, with the larger decreases observed in the intervention group. Sedentary time per day increased by almost 30 min and 10 min in the intervention and control groups, respectively.

**Table 3:** Behavioural and health outcomes at baseline and follow-up

Pre and post- results	Intervention			Control		
	Baseline	Follow-up		Baseline	Follow-up	
Measurement	Mean (SD)	Mean (SD)	Mean change (95% CI)	Mean (SD)	Mean (SD)	Mean change (95% CI)
BMI *z*-score	0.41 (1.16)	0.43 (1.09)	0.02 (− 0.11, 0.15)	0.35 (1.17)	0.41 (1.07)	0.06 (− 0.04, 1.05)
Total daily PA (min)	165 (58)	151 (27)	− 14 (− 87, 115)	144 (41)	143 (42.1)	− 1 (− 117, 121)
Total daily ST (min)	428 (62)	456 (100)	28 (− 174, 120)	407 (81)	417 (52)	10 (− 216, 192)
Total daily steps (count)	12,035 (4084)	10,718 (2020)	− 1316 (− 5818, 8451)	10,221 (3004)	10,017 (3240)	− 204 (− 8235, 8644)

## Discussion

This study investigated the feasibility of a cluster randomised controlled trial of the adapted ToyBox-Scotland childhood obesity prevention intervention. Participating preschools were willing to be randomised, and trial procedures and pre-school-based intervention components were deemed feasible by preschool staff. The intervention was implemented with high fidelity within the preschool. However, implementation of the home component was lower, a finding that is commonly reported in other school-based interventions with home components [32, 35]. The cluster-level recruitment rate of 10% in this study is lower than that achieved in similar feasibility studies targeting young children [36–38], as was the observed individual-level recruitment rate of 18% [36, 37, 39, 40]. Conversely, the overall trial retention rate of 86% (14% attrition rate) is similar to or higher than other trials [6, 36]. However, within those participants that were retained from baseline to follow-up, the collection of valid measures varied considerably by the outcome.

At 41%, the level of implementation observed in the home environment was low. Additionally, the low post-intervention survey response rate of 15% indicates that implementation was even lower, as it is unlikely that non-respondents engaged highly with the intervention. These findings are unsurprising, considering the home environment has previously been identified as one of the more challenging settings to implement obesity prevention interventions in [41], particularly in low SES groups. While overall preschool intervention fidelity was high, it was apparent from logbook responses that PA components of the intervention were implemented at a higher level than SB components (Table 2). This finding was also observed in the original ToyBox study, where SB implementation scores were relatively low across multiple intervention sites within the six participating regions [35]. Considering these findings, we adapted the programme accordingly, reducing the number of more time-consuming activities in the intervention [14]. Despite this, the relatively poor implementation scores observed for SB activities highlights that further adaptation may be needed for the SB component and for the home-based components as a whole.

In the Belgian ToyBox study cohort, recruitment involved a personal visit by a member of research staff to all eligible preschools (*n* = 97), which resulted in a cluster-level recruitment rate of 28% [6]. Additionally, the study achieved an individual-level recruitment rate of 39%, utilising the same procedures of staff-administered information sheets and consent forms to parents as our study. However, it is important to consider the differences in demographics recruited between the two studies. The Belgian study recruited participants from 27 preschools, for which 55% were classed as either medium or high SES. In our study, all but 1 of the 6 recruited preschools were within the 20% most deprived areas in Scotland. An abundance of research has demonstrated that more deprived population groups are more difficult to recruit into trials and are also more likely to drop out than participants in higher SES groups [42–44]. Therefore, the lack of medium-high SES preschools recruited to this present trial may have negatively impacted on the recruitment rates achieved. Considering these observations, in any future trial, it may be of benefit to conduct personal visits to eligible preschools to improve the school recruitment rates, and also target preschools in areas of high, medium, and low SES, using different strategies to recruit participants from deprived populations to account for the lower recruitment rates observed within these areas.

Eighty-six percent of the original sample completed height and weight measures in this study, which is comparable to anthropometric measurement rates of similar studies [7, 39, 45, 46], indicating that these procedures are feasible with this population group. However, we encountered significant issues with the collection of valid BIA data at baseline, and measurement of this outcome was not carried forward to the follow-up. Obtaining accurate BIA readings requires participants to follow a strict protocol consisting of a period of fasting and restricted PA prior to and during the collection of the readings, which was not possible with this sample. Furthermore, there are conflicting arguments in the literature regarding the validity of such methods with children [47]. Our intended use of BIA was to further validate BMI *z*-scores with another measure, as BMI is a crude proxy measure for adiposity [48, 49]. Therefore, in any future trial, it may be beneficial to use other anthropometric measures alongside BMI such as waist circumference, skinfold thickness or hip-waist ratio which have proved feasible in other trials [6, 45], and show high agreement with BMI estimates.

With regard to accelerometry, a number of factors prevented the collection of valid wear-time data at both baseline and follow-up. Studies that use objective measures of free-living PA and SB in children offer conflicting findings with regards to compliance with measurement procedures. A recent review of attrition rates and non-compliance with accelerometers in childhood PA trials found that non-compliance at follow-up ranged from 3 to 70% across 23 studies [50]. Conversely, Jones et al. (2011) used Actigraph accelerometers worn for two consecutive days in a pilot RCT of a fundamental movement skills and PA intervention in preschool children, reporting high adherence rates of 96% and 97% for baseline and 6-month follow-up, respectively [51], indicating that reduced wear-time may increase compliance. However, a similar study used the Actigraph for 7 days in pre-schoolers and achieved an 86% adherence rate, indicating additional factors likely influence accelerometer compliance [51].

While some unavoidable reasons for loss of data such as device malfunction/loss and child absence in our study reflect issues commonly encountered in accelerometer studies mentioned previously, a number of reasons for the early removal of the device are specific to the activPAL accelerometer that we used. Specifically, a small number of parents reported that their child developed a rash due to wearing the waterproof medical adhesive which attached the device to the leg. While this was likely a harmless sweat rash, a recent study also found that adolescents who were asked to record their reasons for removing the activPAL in a compliance study cited skin irritation as the primary reason for early removal [53], a finding which is supported by another study on pre-schoolers [24]. These issues could be addressed by reducing the required wear time and improving communication with parents so that they are aware of how to reattach the device or how to distinguish a sweat rash from an allergic reaction. Alternatively, the activPAL showed to be comparable with other wearable devices that are perhaps less invasive and more participant-friendly in their attachment methods [24, 27] which may be valid alternatives in further trials. Regardless of the device used, creating better links with parents from recruitment through to follow-up will likely facilitate the collection of a higher proportion of valid accelerometer data and should be a priority in any future trial. A number of trials have reported favourable results from the use of reminder texts/phone calls to parents or the provision of small monetary incentives for the safe return of accelerometers [39].

Parental response rates to the FFQ and PCQ questionnaires were also considerably lower than rates observed in the original ToyBox study and in other trials [54]. Although efforts to reduce the length of these questionnaires were taken prior to the trial commencing, additional adaptation may be needed to increase the response rates. Due to the need to calculate portion sizes and recall dietary patterns, the FFQ can be time-consuming to complete [55, 56], and parents may not know what their child has eaten while at preschool. This, coupled with the relatively low levels of adult literacy observed in the areas which we sampled [57], may have negatively impacted our questionnaire response rates. Therefore, other more time-efficient alternatives to the FFQ should be explored [56].

This study had a number of limitations. Firstly, due to the low preschool-level response rate, it was only possible to sample preschools located in areas of lower SES, which limits the generalisability of our findings to the wider Scottish preschool population. However, this issue is somewhat unavoidable when conducting research within Glasgow, which has a significantly higher concentration of deprived localities than the rest of Scotland’s local authority areas [58]. This issue could be addressed in the future through the use of stratified sampling, which would allow for the assessment of differences in intervention effectiveness between SES groups. Despite this, due to the marked social patterning observed in obesity risk, there is a need to target interventions at low SES groups [59]; therefore, the lessons learned from this study will be of value during the design of future trial procedures.

Secondly, while the aim of this study was to test feasibility, and not to test effectiveness, the low response rates to questionnaires and non-compliance with accelerometer measures makes it difficult to determine any direction of intervention effect, which would have indicated whether the intensity of the intervention was likely to be sufficient. Despite these limitations, the data gathered during this trial are sufficient to assess the feasibility of the study design and the fidelity of the intervention, which will assist with the development of effectiveness and efficacy trials. Another important aspect of feasibility testing not addressed in this present paper is the acceptability of the intervention and trial procedures. Items pertaining to acceptability were included in both teacher and parent post-intervention questionnaires, and qualitative interviews and focus groups were conducted with both parents and practitioners, respectively. A separate paper will present these data and explore acceptability by identifying important barriers and facilitators to implementation.

## Conclusions

The findings of this study indicate that although aspects of this cluster RCT of the ToyBox Scotland intervention were feasible, more efforts to increase recruitment rates, accelerometer compliance, and questionnaire response rates should be further investigated before progression to any future trial. Specifically, more development activities should be undertaken with preschools, parents, and children to ensure that both the intervention components and the methods of evaluation are appropriate and acceptable before progressing to further effectiveness testing.

Testing feasibility before progressing to a fully powered trial is an effective way to identify issues with sampling/recruitment, implementation fidelity, trial design, and methods of outcome measurement. This study, coupled with the results of an ongoing investigation of the barriers and facilitators to implementation of the intervention, will further highlight priorities for further adaptation prior to any future trial of the ToyBox Scotland intervention.

## Supplementary information

**Additional file 1.** The ToyBox preschool health programme parent/guardian feedback form.

## Data Availability

As data were collected from local authority preschool children, public availability of the datasets is restricted by Glasgow City Council data protection policies. However, datasets relating to this study are available from the corresponding author upon reasonable request.

## Abbreviations

BIA: Bioelectrical impedance analysis
BMI: Body mass index
FFQ: Food frequency questionnaire
LDL: Low-density lipoprotein
PA: Physical activity
PCQ: Primary caregiver questionnaire
SB: Sedentary behaviour
SES: Socioeconomic status
ST: Sedentary time
WHO: World Health Organisation
