# Supplementary material for: A feasibility cluster randomised controlled trial of a preschool obesity prevention intervention: ToyBox-Scotland
Source: Pilot Feasibility Stud. 2019 Nov 9;5:128. doi: 10.1186/s40814-019-0521-7 (PMC6842492; doi:10.1186/s40814-019-0521-7)
Supplement: Supplementary file 1 — Additional file 1. The ToyBox preschool health programme parent/guardian feedback form. [file 40814_2019_521_MOESM1_ESM.pdf]

The ToyBox preschool health programme- parent/guardian feedback form

The questions below ask about the materials that accompanied the ToyBox programme. Please tick **one** box for each question	**Yes**	**No**	**Not sure**
Did you receive the ToyBox-Scotland sticker wallchart from your child's preschool?			
If so, did you use the wallchart?			
Did you receive the home activity pack called "fun in the forest"?			
Did you receive the home activity pack called "leaping at the loch"?			
Did you receive the home activity pack called "fun on the farm"?			
Did you receive the home activity pack called "adventures at loch ness"?			
Did you receive the home activity pack called "moving in the mountains"?			
Did you receive the home activity pack called "flying with the eagles"?			
Did you receive the home activity pack called "Fox's games"?			

The next two questions are about your use of the ToyBox home materials. Please tick **one** box for each question	**None**	**Some**	**Not sure**	**Most**	**All**
How many of the ToyBox home activities that you received did you use at home with your child?					
Did you award the provided stickers to your child after they completed an activity?					

The next questions are about your feelings towards the ToyBox home materials. Please tick **one** box for each question	**Strongly disagree**	**disagree**	**Not sure**	**agree**	**Strongly agree**
Overall, did your child enjoy the activities in the programme?					
Overall, did your child like the stickers and wallchart provided?					
Did you enjoy doing the activities with your child?					
Do you think the activities helped your child be more physically active?					
Do you think the activities helped your child spend less time sitting/being inactive?					
Do you think the activities helped your child to eat healthier snacks?					
Do you think the activities helped your child drink more water instead of sugary juices?					
Were the instructions provided for the games/activities easy to read and clear?					

Please provide any additional comments that you would like to make about the ToyBox home materials and activities in the box below:

Thank you for taking the time to complete this feedback form!
